# Traumatic diaphragmatic injuries: epidemiological, diagnostic and therapeutic aspects

**DOI:** 10.1186/s40064-016-3291-1

**Published:** 2016-09-20

**Authors:** Ousmane Thiam, Ibrahima Konate, Mohamadou Lamine Gueye, Alpha Omar Toure, Mamadou Seck, Mamadou Cisse, Balla Diop, Elias Said Dirie, Ousmane Ka, Mbaye Thiam, Madieng Dieng, Abdarahmane Dia, Cheikh Tidiane Toure

**Affiliations:** 1 General Surgery Department, Aristide Le Dantec Teaching Hospital, Dakar, Senegal; 2 Surgery and Surgical Specialties Department, Gaston Berger University, Saint-Louis, Senegal

**Keywords:** Diaphragmatic injury, Diaphragmatic hernia, Blunt abdominal trauma, Blunt chest trauma, plaie diaphragmatique, hernie diaphragmatique, contusion abdominale, contusion thoracique

## Abstract

**Introduction:**

Diaphragmatic injuries include wounds and diaphragm ruptures, due to a thoracoabdominal blunt or penetrating traumas. Their incidence ranges between 0.8 and 15 %. The diagnosis is often delayed, despite several medical imaging techniques. The surgical management remains controversal, particularly for the choice of the surgical approach and technique. The mortality is mainly related to associated injuries. The aim of our study was to evaluate the incidence of diaphragmatic injuries occuring in thoraco-abdominal traumas, and to discuss their epidemiology, diagnosis and treatment.

**Patients and methods:**

We performed a retrospective study over a period of 21 years, between January 1994 and June 2015 at the Department of General Surgery of the Aristide Le Dantec hospital in Dakar, Senegal. All patients diagnosed with diaphragmatic injuries were included in the study.

**Results:**

Over the study period, 1535 patients had a thoraco-abdominal trauma. There were 859 cases of blunt trauma, and 676 penetrating chest or abdominal trauma. Our study involved 20 cases of diaphragmatic injuries (1.3 %). The sex-ratio was 4. The mean age was 33 years. Brawls represented 83.3 % (17 cases). Stab attacks represented 60 % (12 cases). The incidence of diaphragmatic injury was 2.6 %. The wound was in the thorax in 60 % (seven cases). Chest radiography was contributory in 45 % (nine cases). The diagnosis of wounds or ruptures of the diaphragm was done preoperatively in 45 % (nine cases). The diaphragmatic wound was on the left side in 90 % (18 cases) and its mean size was 4.3 cm. The surgical procedure involved a reduction of herniated viscera and a suture of the diaphragm by “X” non absorbable points in 85 % (17 cases). A thoracic aspiration was performed in all patients. Morbidity rate was 10 % and mortality rate 5 %.

**Conclusion:**

The diagnosis of diaphragmatic rupture and wounds remains difficult and often delayed. They should be kept in mind in any blunt or penetrating thoraco-abdominal trauma. Diaphragmatic lesions are usually located on the left side. Surgery is an efficient treatment.

## Résumé

**Introduction:**

Les traumatismes du diaphragme comprennent les ruptures et les plaies du diaphragme. Leur incidence varie entre 0,8 % et 15 %. Elles sont très souvent méconnues malgré les techniques performantes d’imagerie médicale. Leur prise en charge chirurgicale reste controversée. La mortalité de cette pathologie est liée aux lésions associées. Le but de notre étude était d’apprécier l’incidence des lésions diaphragmatique dans les traumatismes thoraco-abdominaux, et de discuter les aspects épidémiologiques, diagnostiques et thérapeutiques.

**Patients et méthode:**

Il s’agissait d’une étude rétrospective sur 21 ans allant du 1^er^ janvier 1994 au 30 juin 2015. Cette étude a été réalisée au Service de Chirurgie Générale de l’Hôpital Aristide Le Dantec de Dakar. Etaient inclus dans cette étude tous les patients qui présentaient une lésion diaphragmatique consécutive à un traumatisme abdominal et/ou thoracique ouvert ou fermé.

**Résultats:**

Durant cette période d’étude, nous avons reçu 1535 patients victimes de traumatisme thoracique et/ou abdominal. Il s’agissait de 859 cas de contusions et 676 cas de plaies thoraciques et/ou abdominaux. Notre étude portait sur 20 cas de lésions diaphragmatiques (1,3 %). Le sex-ratio était de 4. L’âge moyen était de 33 ans. Les agressions par arme blanche représentaient 60 % (12 cas). L’incidence des lésions diaphragmatiques était de 2,6 %. La plaie cutanée était de siège thoracique dans 60 % (7 cas). La radiographie du thorax était contributive dans 45 % (9 cas). Le diagnostic de lésion diaphragmatique était préopératoire dans 45 % (9 cas). La brèche diaphragmatique siégeait à gauche dans 90 % (18 cas) et la taille moyenne était de 4,3 cm. Le geste chirurgical avait consisté en une réduction des viscères herniés et une suture du diaphragme par des points en « X » dans 85 % (17 cas). Le drainage thoracique était systématique. Le taux de morbidité était de 10 % et la mortalité de 5 %.

**Conclusion:**

Leur diagnostic est difficile. Elles siègent le plus souvent à gauche. Leur traitement est chirurgical et la voie d’abord préférentielle est la laparotomie.

## Background

Diaphragmatic injuries include wounds and diaphragm ruptures, due to a thoracoabdominal blunt or penetrating traumas. They occur in a context of multiple trauma (Bosanquet et al. 2009). Their diagnosis can be done early, but they are very often ignored, despite performing medical imaging techniques (Waldschmidt and Laws 1980). When they are missed, diagnosis is often late when there is a complication. Their incidence goes between 0.8 and 1.6 % for abdominal contusion, and between 10 and 15 % in chest wounds (Epstein and Lempke 1968; Reber et al. 1998). Their diagnosis is difficult. The surgical treatment is controversal, particularly for the surgical approach and techniques. The mortality is mainly related to associated injuries. The aim of our study was to evaluate the incidence of diaphragmatic injuries in the thoracic-abdominal trauma and discuss the epidemiology, diagnosis and treatment.

## Patients and methods

We performed a retrospective study over a period of 21 years, between 1th January 1994 and 30 June 2015. This study was conducted in General Surgery Department at Aristide Le Dantec hospital in Dakar. Were included in this study, all patients diagnosed with diaphragmatic injuries.

## Results

**Fig. 1:**
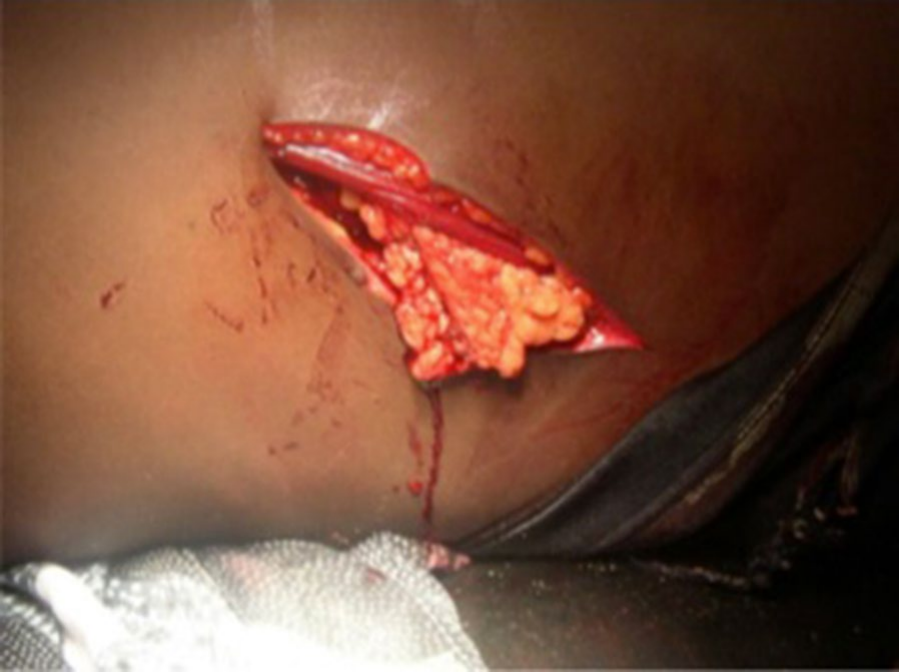
Large left thoraco-abdominal wound with epiplocele

**Fig. 2:**
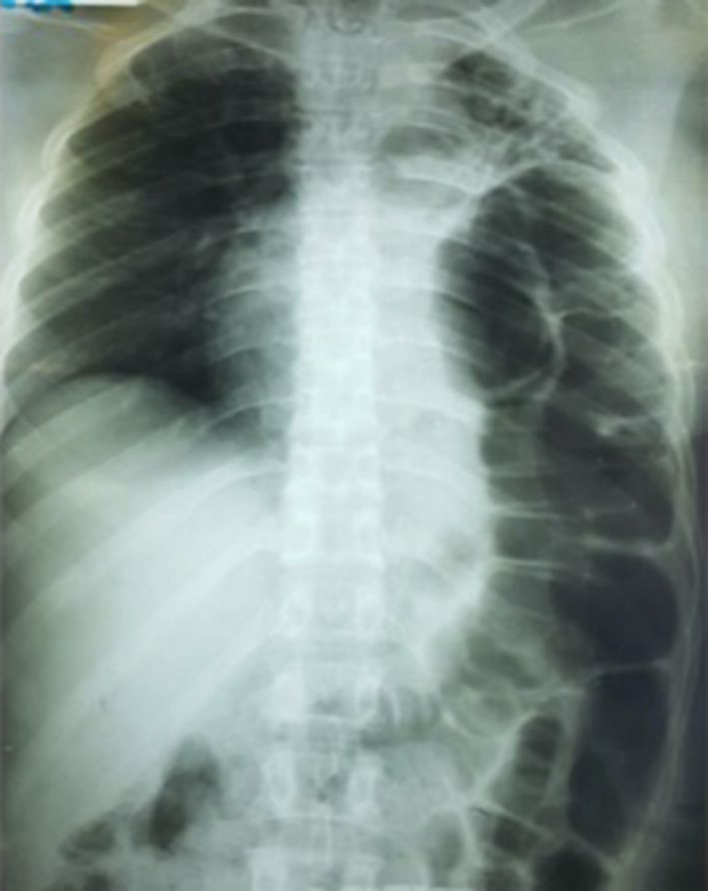
Abdominal x-rays showing left diaphragmatic hernia

**Fig. 3:**
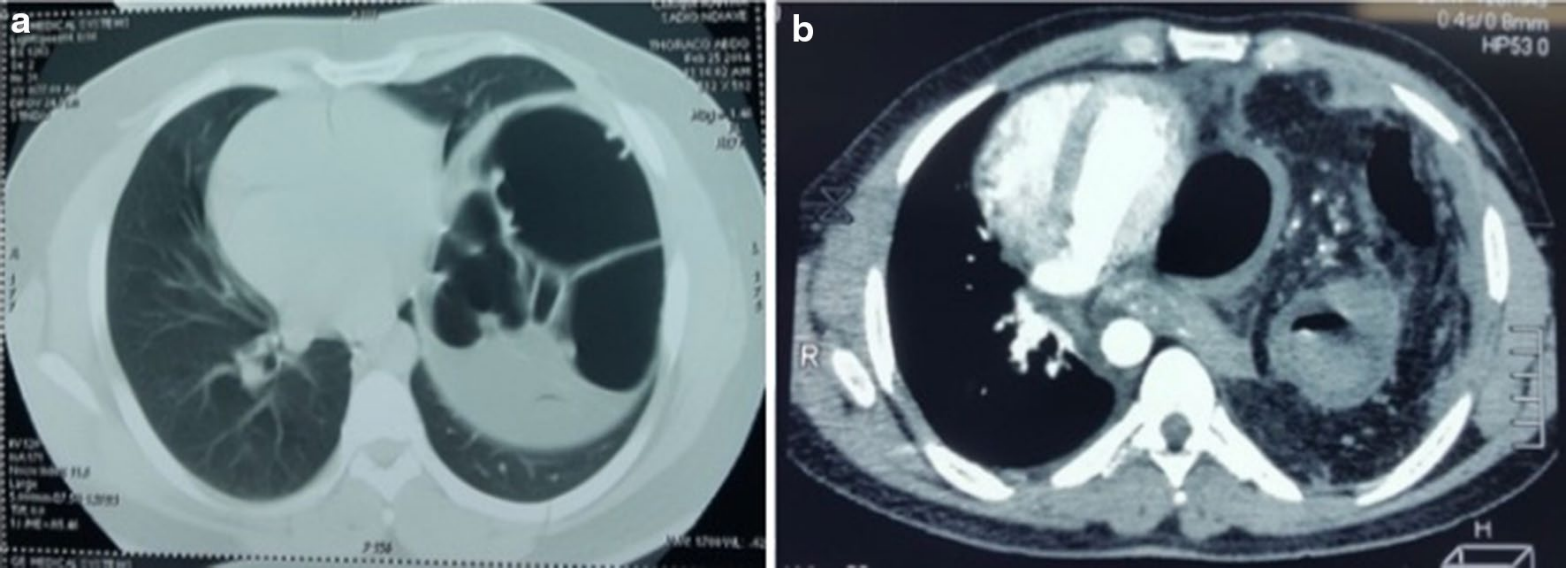
**a**, **b** Chest CT scan showing a left diaphragmatic hernia

**Fig. 4:**
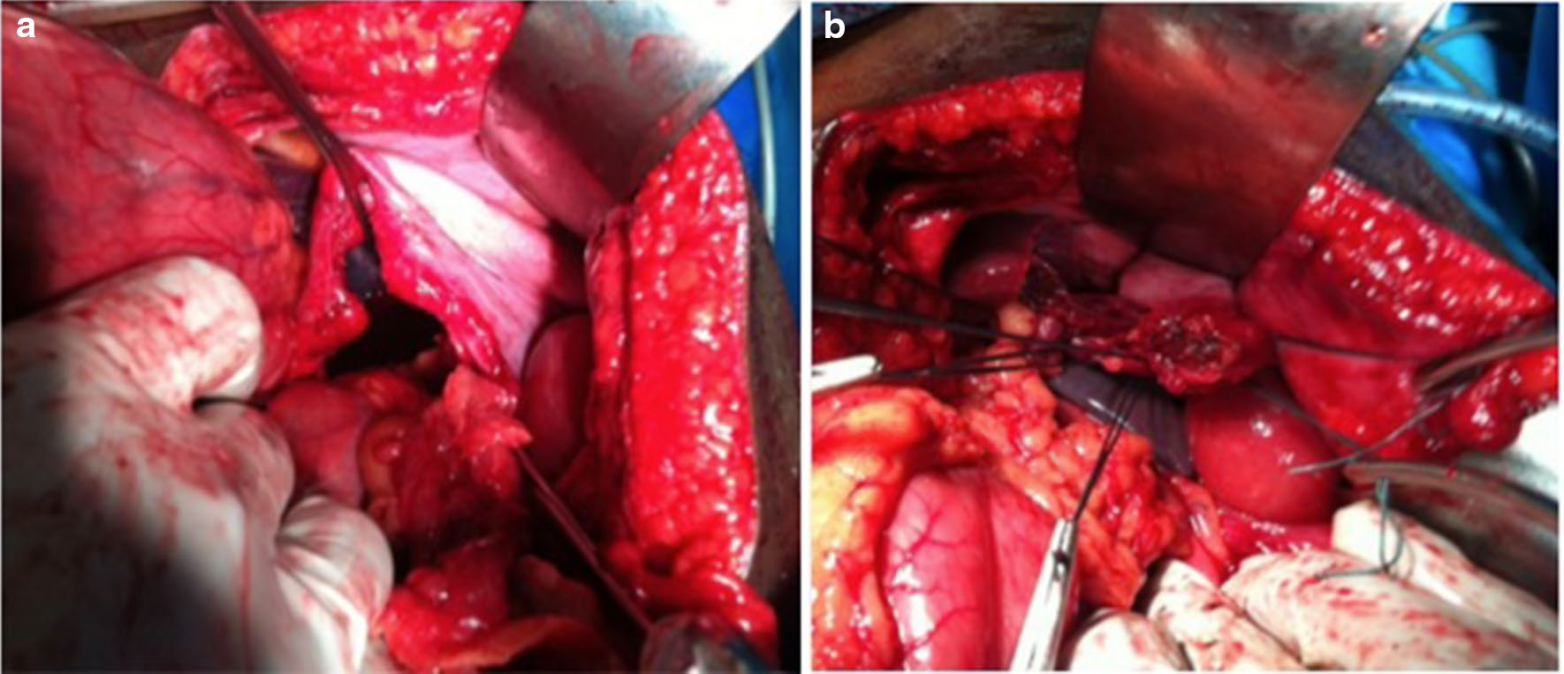
Intraoperative view of a left diaphragmatic rupture before (**a**) and after repair (**b**)

During the study period, 1535 patients were admitted for chest and/or abdominal trauma. There were 859 cases of blunt trauma, and 676 penetrating chest or abdominal trauma. Our study included 20 cases (1.3 %) of diaphragmatic injuries. They were 16 men and 4 women with a sex ratio of 4. The average age was 33 years, with extremes of 20 and 40 years. For 19 patients, the mean time to admission was 2.4 days with extremes of 5 h and 21 days. For one patient, the admission’s period was 1 year after a chest stab wound drained. The circumstances were a brawl in 17 cases (83.3 %) and a traffic accident in 3 cases (16.7 %). The mechanism was a stab attack in 12 cases (60 %), thoracoabdominal contusion in 6 cases (30 %) and a gunshot wounds in 2 cases (10 %). The incidence of diaphragmatic injury was 0.2 % in contusions and 2.6 % in abdominothoracic penetrating wounds. The wound was thoracic in seven cases (60 %), abdominal in three cases (30 %) and thoracoabdominal in one case (10 %). The average length of the wound was 3.8 cm, with a range of 1.5–9 cm (Fig. 1). Chest radiography performed in all patients was contributory in 9 cases (45 %). It showed a supra-diaphragmatic digestive clarity in seven cases, hydro-pneumothorax in one case and an elevated left hemi-diaphragm in one case (Fig. 2). An abdomen x-ray was performed in seven patients and showed one case of pneumoperitoneum. The abdominal ultrasound, perfomed in all patients with abdominal contusions (six cases), was normal in five cases and showed a splenic wound and abdominal effusion in one case. The thoracoabdominal CT scan performed in three patients, showed diaphragmatic hernia in all cases (Fig. 3a, b). The diagnosis of diaphragmatic hernia was made before surgery in nine cases (45 %), during surgery in ten cases (50 %) and at autopsy in one case (5 %). Surgical approach was a laparotomy in 16 cases (80 %), a thoracotomy in 2 cases (10 %) and a laparoscopy in 5 % (1 case). The anatomic distribution of injury to the diaphragm included 18 left-sided injuries (90 %) and 2 right-sided injuries (10 %). The mean sizes of the defects was 4.3 cm with a range of 1.5 and 12 cm. Herniated viscera were: stomach (one case), small bowel (one case) and epiploon (two case). Associated injuries were: one gastric perforation, two splenic wounds, one liver wound, three pelvic fractures, one scapular belt fracture, four rib fractures and one L1 and L3 transverse process fracture. The surgical procedure consisted in a reduction of herniated organs, repair associated lesions and a suture of the diaphragm with the “X” non absorbable points in 80 % (16 cases) and «paletot» suture in 15 % (n = 3) (Fig. 4a, b). The chest drainage was done in all patients. The mean duration of thoracic drainage was 3 days, with extremes of 2 days and 8 days. The mean hospital stay was 6 days with extreme of 4 and 10 days. Mortality rate was 5 %. One patient died of acute respiratory distress. Morbidity rate was two cases (10 %). It was one case of lung atelectasis, with uneventful course. One case of recurrence was noted 9 months after diaphragmatic laparoscopical suture. It was treated with a composite prosthesis by open surgery.

## Discussion

**Fig. 5:**
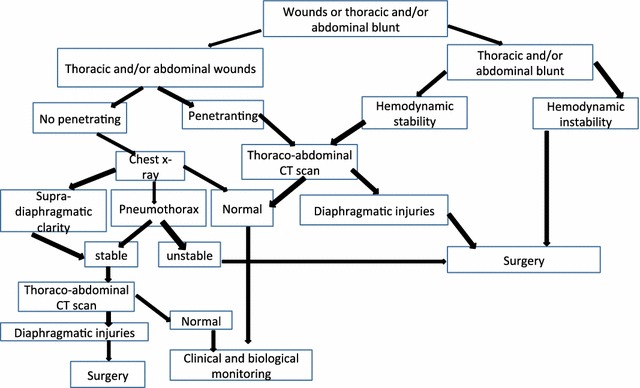
Algorithm of dioagnosis and treatment

Diaphragmatic injuries are observed in violent trauma. During the last decade, there has been an increase in industrialized countries (Duverger et al. 2001; Moreaux and Perrotin 1965). In our study, diaphragmatic injuries represented 1.3 % of all chest and/or abdomen trauma. Its real impact is not very well known in our regions because there are other emergency units. Rubikas et al. reported an incidence of 2.1 % of diaphragmatic trauma in patients with thoracoabdominal contusion, and 3.4 % in penetrating trauma wich is higher to our study (Rubikas 2001). But it is significantly higher in penetrating wounds of 7 versus 3.4 % (Rubikas 2001). In our series, the incidence of diaphragmatic injury in penetrating wound is close to North American studies (Moore et al. 1994; Beal and Mc Kennan 1988). Our results are different to those of Shah et al., who reported a diaphragmatic injury rates by 75 % in thoracoabdominal contusion and 25 % in penetrating trauma (Shah et al. 1995; Fair et al. 2015). Diaphragmatic injuries involve 1–7 % of thoracoabdominal contusions and 10–15 % of chest wounds (Reber et al. 1998; Igai et al. 2007). The incidence of diaphragmatic injury is often underestimated in over half the cases, especially those located at right side (Reber et al. 1998; Shah et al. 1995). In our study, the right diaphragmatic injury represented two cases (10 %). This rate is similar to those found in the literature, ranging between 5 and 30 % of all thoracic and/or abdominal trauma (Wirbel and Mutschler 1998; Sukul et al. 1991; Rodrigues-Morales et al. 1986). Diaphragmatic injuries often occur in a context of multiple trauma and are often associated with pelvic, chest wall and members lesions as in our series (Reber et al. 1998; Wirbel and Mutschler 1998; Sukul et al. 1991; Boulanger et al. 1993). Diaphragmatic injuries are found among young males, as it was the case in our study (Shah et al. 1995; Sukul et al. 1991; Athanassiadi et al. 1999). The diagnosis can be suspected on clinical examination with air-fluid noises in the chest. Sukul et al. had the diagnosis done on clinical examination in 14 % of cases. In our study, chest radiography was contributory to diagnosis of wounds and diaphragmatic rupture in 45 % of cases (Sukul et al. 1991). Its diagnostic value was thus significantly higher than that reported in Sukul et al. study, which was 21 % (Sukul et al. 1991). Abdominal CT scan has a good diagnostic sensitivity in wounds and diaphragmatic ruptures, but it must be done on stable patients (Mihos et al. 2003). The best way to make the faster diagnosis of diaphragmatic injury, is to evocate it systematically before any contusion and/or thoracoabdominal penetrating wound. According to Shapiro et al, was done preoperatively in 43.5 % of cases, intraoperatively or during an autopsy in 41.3 % of cases, then later after the injury in 14.6 % of cases (Shapiro et al. 1996). In our study, the diagnosis was preoperative in 33.3 % of cases, intraoperative in 60 % of cases and found an autopsy in 6.7 % of cases. In the study of Muray et al., 24 % were discovered during surgery or after an autopsy (Murray et al. 2001). The choice of the surgical approach is controversal, due to the non-operative therapies approach and minimally invasive surgery. However, laparotomy is admited unanimously by all authors to the urgent exploration of wounds and thoracoabdominal contusions (Sukul et al. 1991; Beauchamp et al. 1984). Laparotomy approach can dignosis and take care of associated lesions. Thoracotomy is indicated in the case of late diaphragmatic hernia, isolated lesions of the right diaphragm and in case of suspicion of chest injury. Diaphragmatic injuries was considered chronic if the diagnosis was delayed from the trauma. Our diagnostic and therapeutic strategy is summarized in the algorithm (Fig. 5). No surgical complications were found in our study. However, diaphragmatic paralysis can be found (Sukul et al. 1991). Mortality is high and may reach 20 % (Fair et al. 2015; Sukul et al. 1991). It is often related to associated injuries. In our series, the death rate was 5 %.

## Conclusion

The diagnosis of wounds and diaphragmatic rupture remains difficult and often delayed. They should be kept in mind in any blunt or penetrating thoraco-abdominal trauma. Diaphragmatic lesions are usually located on the left side. Surgery is an efficient treatment.
